# Layer by Layer Ex-Situ Deposited Cobalt-Manganese Oxide as Composite Electrode Material for Electrochemical Capacitor

**DOI:** 10.1371/journal.pone.0129780

**Published:** 2015-07-09

**Authors:** Rusi, P. Y. Chan, S. R. Majid

**Affiliations:** Centre for Ionics University of Malaya, Department of Physics, Faculty of Science, University of Malaya, Kuala Lumpur, Malaysia; Institute for Materials Science, GERMANY

## Abstract

The composite metal oxide electrode films were fabricated using ex situ electrodeposition method with further heating treatment at 300°C. The obtained composite metal oxide film had a spherical structure with mass loading from 0.13 to 0.21 mg cm^-2^. The structure and elements of the composite was investigated using X-ray diffraction (XRD) and energy dispersive X-ray (EDX). The electrochemical performance of different composite metal oxides was studied by cyclic voltammetry (CV) and galvanostatic charge-discharge (CD). As an active electrode material for a supercapacitor, the Co-Mn composite electrode exhibits a specific capacitance of 285 Fg^-1^ at current density of 1.85 Ag^-1^ in 0.5M Na_2_SO_4_ electrolyte. The best composite electrode, Co-Mn electrode was then further studied in various electrolytes (i.e., 0.5M KOH and 0.5M KOH/0.04M K_3_Fe(CN) _6_ electrolytes). The pseudocapacitive nature of the material of Co-Mn lead to a high specific capacitance of 2.2 x 10^3^ Fg^-1^ and an energy density of 309 Whkg^-1^ in a 0.5MKOH/0.04MK_3_Fe(CN) _6_ electrolyte at a current density of 10 Ag^-1^. The specific capacitance retention obtained 67% of its initial value after 750 cycles. The results indicate that the ex situ deposited composite metal oxide nanoparticles have promising potential in future practical applications.

## Introduction

Electrochemical supercapacitors are appealing as devices for storing electrical energy, because they can deliver power at higher rates than batteries and higher energy density than conventional capacitors [1–3]. They can also be classified by their charge storage mechanism (i.e., as electrical double layer capacitors (EDLC) and pseudocapacitors). The energy storage mechanism of EDLC depends on the accumulated charge at the electrode-electrolyte interface. Pseudocapacitors are being developed in order to improve energy density through the storage mechanism of intercalation/deintercalation cations from the electrolyte into the electrode. In pseudocapacitors, the joint action of non-Faradic double-layer charge storage processes and redox reactions results in high capacitance and energy storage density [4–7]. A lot of materials have been studied as potential electrode materials for supercapacitors including (1) carbonaceous materials, (2) conducting polymers, and (3) transition metal oxides [7, 8]. Out of these materials, metal oxide electrode materials have attracted considerable interest because of their large capacitance and fast redox kinetics. There are some other fields of studies, such as optical, membrane fusion, and magnetron sputtering, that have utilized metal oxides to obtain a higher level of performance [9–12]. Metal oxide materials such as MnO_2_, NiO, Co_3_O_4_, and VO are commonly used as candidate materials for pseudocapacitor electrodes [5–6].

MnO_2_ has received attention because its physical and chemical properties can be used to make relatively high quantities of low-cost, non-toxic pseudocapacitors with a high specific capacitance value [7, 13–14]. However, as a supercapacitor electrode material, MnO_2_ is still hampered by its poor electrical conductivity and material dissolution during electrochemical cycling, which leads to a severe specific capacitance drop as the scan rate increases. To mitigate these problems, many measures such as making nanocomposites and carbon-mixing have been carried out [7, 14]. To obtain nanocomposite compounds, the primary MnO_2_ metal oxide can be incorporated with secondary/ternary metal oxide materials [7, 15]. In this case, an electrode containing mixed metal oxides performs better than a single transition metal oxide when it is used as the electrode in a supercapacitor [15]. For instance, F. Gobal and S. Jafarzadeh [16] employed the deposition method to obtain binary cobalt-manganese oxides on stainless steel substrate, and the ex situ deposited metal oxide showed higher specific capacitance than a single deposited metal oxide. However, the morphology changes and the effect of the second layer have not been studied. According to Lee et al. [17], binary manganese-nickel (Mn-Ni) oxide films in situ electrodeposited from a bath consisting of manganese acetate and nickel chloride on a graphite sheet can achieve a specific capacitance of 424 Fg^-1^ in Na_2_SO_4_ electrolyte at a scan rate of 20 mVs^-1^. In the work of Prasad and Miura [18], the addition of cobalt oxide showed an improved specific capacitance of the manganese oxide electrode. The purpose of this work is to investigate the electrochemical characteristics of a composite MnO_2_-based electrode that has been prepared by the ex situ electrodeposition technique. We have chosen to use the electrodeposition technique due to several advantages provides, such as being simple to set up, requiring low deposition temperature, allowing for easy control of the deposited thickness, consuming less energy, and being fast to be carry out [19]. A layer of MnO_2_ was used as a first layer, followed by the ex situ deposition of a second layer containing mixed metal hydroxide solutions, such as Co(OH)_2_, NiOH, and Mn(OH)_2_, performed according to the chronopotentiometry method. The influence of the second layer on the morphological structure and the electrochemical performance of the best electrode tested in different electrolytes are also discussed within this study.

## Experimental

### Materials

In this work, manganese acetate tetrahydrate (Mn(CH_3_COO)_2_.4H_2_O) and nickel acetate tetrahydrate, (Ni(CH_3_COO)_2_.4H_2_O), were purchased from Aldrich and used without further purification. Cobalt sulphate (CoSO_4_.7H_2_O) and sulphuric acid H_2_SO_4_ were obtained from Unilab and Friendemann Schmidt, respectively.

### Electrodeposition

The electrodeposition was conducted under ambient conditions at 27°C with a three-electrode configuration in chronopotentiometry mode using an Autolab PGSTAT30. Stainless steel (SS), Ag/AgCl and carbon rod were respectively used as the working, reference and counter electrodes. The area of the working electrode in contact with the solution was fixed at 4 cm^2^. The first layer was made up of manganese hydroxide particles electrodeposited from a 0.01M of Mn(CH_3_COO)_2_.4H_2_O aqueous solution for 300s [20]. The current density was fixed at 2 mAcm^-2^ and the obtained sample was further heated at 150°C for 6 hours. The second layer was electrodeposited on the first layer after the sample was cooled to 27°C. The deposition current density was fixed at 2 mA cm^-2^ for 300s using the different deposition solutions listed in Table 1.

**Table 1:** List of deposition solutions for the second layer.

Sample	Deposition solution
Co-Mn	6ml of 0.8 M of H_2_SO_4_+30 ml of 0.01M Mn(CH_3_COO)_2_.4H_2_O+30 ml 0.15M CoSO_4_.7H_2_O
Ni-Mn	6ml of 0.8M H_2_SO_4_+30ml of 0.01M Mn(CH_3_COO)_2_.4H_2_O+30ml of 0.25M Ni(CH_3_COO)_2_.4H_2_O
Co-Ni	6ml of 0.8M H_2_SO_4_+30 ml 0.15M CoSO_4_.7H_2_O+30ml of 0.25M Ni(CH_3_COO)_2_.4H_2_O
Co-Ni-Mn	6ml of 0.8M of H_2_SO_4_+20ml of 0.01M Mn(CH_3_COO)_2_.4H_2_O+20ml of 0.25M Ni(CH_3_COO)_2_.4H_2_O+20 ml 0.15M CoSO_4_.7H_2_O

Lastly, all of the prepared electrodes were rinsed with distilled water and heated at 300°C for 6 hours before further characterization. The sample preparation and measurements are reproducible.

### Characterization

The morphology of the deposited film was investigated using field emission scanning electron microscopy (FeSEM), Joel JSM-7600F, and transmission electron spectroscopy (TEM) Jeol JEM-2100F. The crystal structure of deposited metal oxide and powder from the selected sample scraped off the SS was examined by an X-ray diffraction (XRD) D8 Advance X-Ray diffractometer-Bruker AXS using CuK_α_ monochromatized radiation at 40 kV and 40 mA at ambient temperature. The electrochemical performance was examined using electrochemical impedance spectroscopy (EIS), cyclic voltammetry (CV) and charge-discharge (CD) studies. All the electrochemical tests were set up with three electrode systems: prepared electrode as working electrode, platinum as a counter electrode, and Ag/AgCl as a reference electrode. The electrochemical impedance spectroscopy (EIS) tests were performed at a frequency of 0.1Hz to 100 kHz at applied AC potential of 0 V.

## Results and Discussion

### Morphological and structural studies

**Fig 1:**
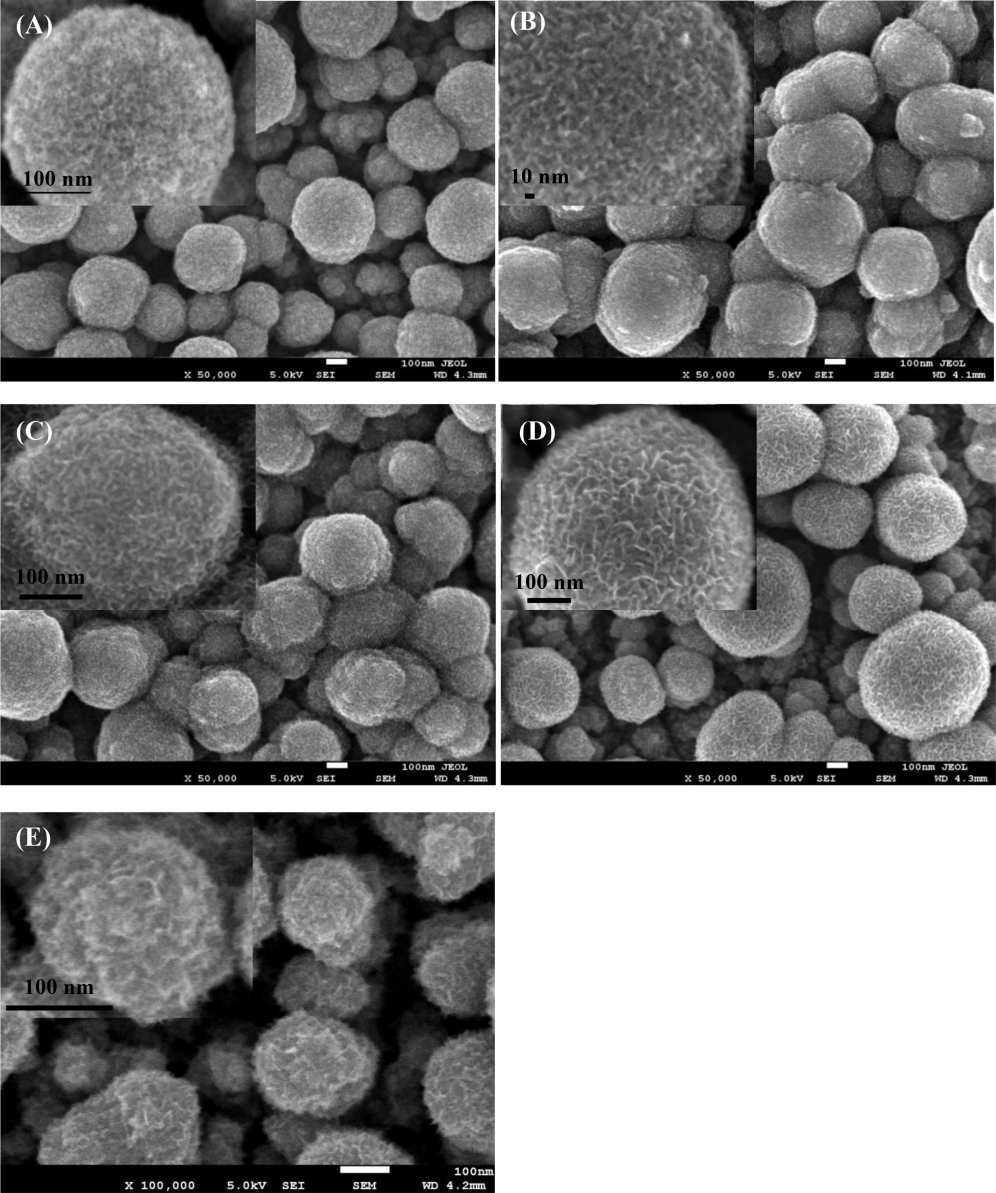
FESEM images at high and low magnification: (A) Co-Mn, (B) Ni-Mn, (C) Co-Ni-Mn, (D) Co-Ni, and (E) first layer of deposited MnO_2_.

**Fig 2:**
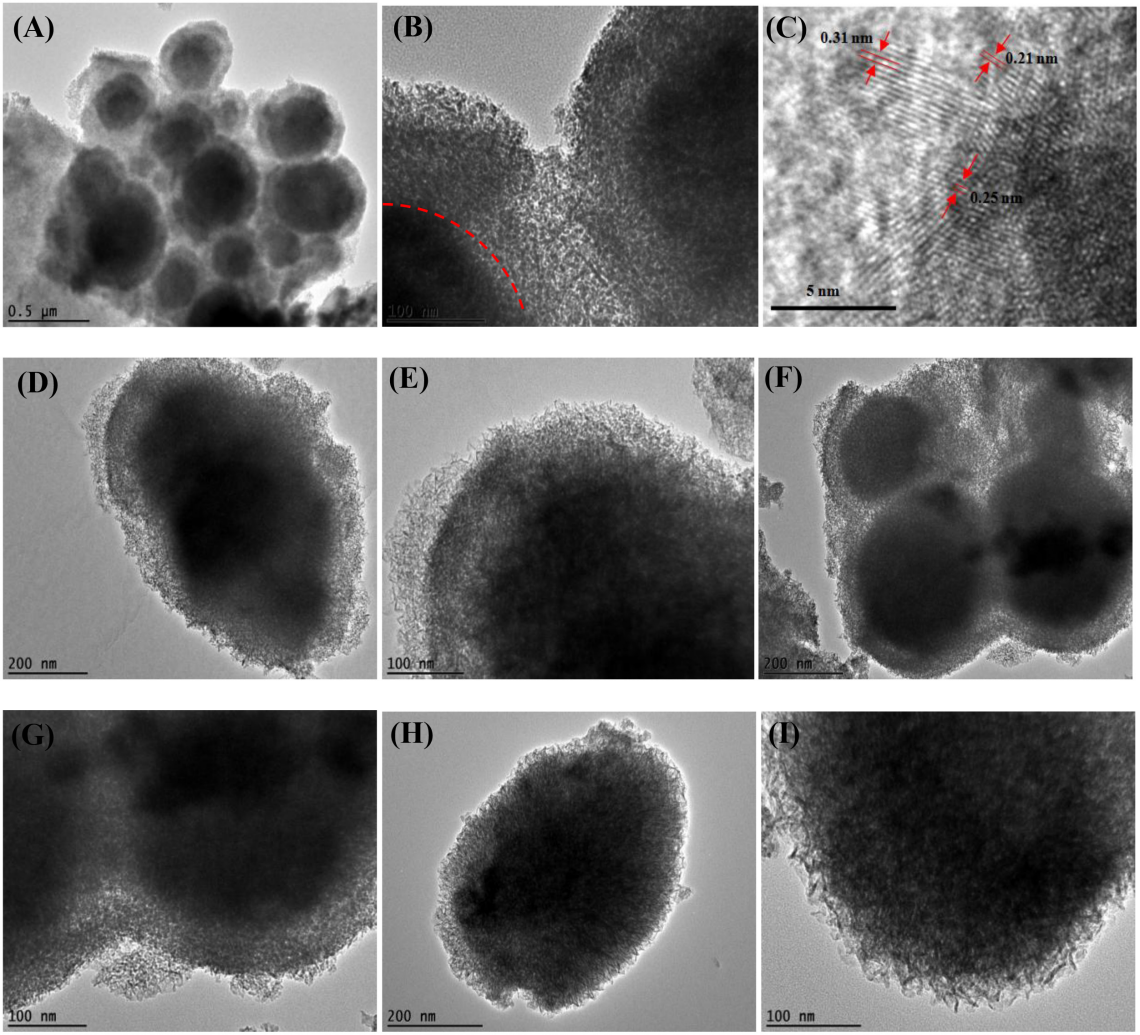
TEM images at low and high magnification: (A-B) Co-Mn, (C) inter-planar lattice spacing of Co-Mn, (D-E) Ni-Mn, (F-G) Co-Ni-Mn, and (H-I) Co-Ni.

FESEM and TEM analyses were carried out to probe the surface structural identities of Co-Mn, Ni-Mn, Co-Ni, and Co-Ni-Mn deposited samples. The FESEM images are displayed in Figs 1 and 2. As can be seen from the low magnification image of all samples, most of the deposited particles are spherical in shape. The sizes of the spherical particles are in the range of 100 to 400 nm. The contiguous particles are only observed in the FESEM images of Ni-Mn and Ni-Co-Mn, shown in Fig 1B and 1C, which tended to form large agglomerated particles. The surface of the particles exhibits a nanoflake-like structure, which is influenced by the nucleation process of the second electrodeposited layer. As shown in Fig 2, higher magnification of TEM images reveals that a flower-like structure is clearly seen in all samples. The thickest layer of the flower-like structure at the outer particles (40–70 nm) is observed in the Co-Mn sample (Fig 2A and 2B), which may be constructive for ion intercalation. The interplanar spacing of Co-Mn is shown in Fig 2C. The periodic lattice fingers’ distance of 0.21 nm and 0.31 nm might attributed to the interplanar spacing of the (301) and (310) MnO_2_ plane, while 0.25 nm belongs to the (311) plane of Co_3_O_4_ [20–21]. In the deposited Ni-Mn and Ni-Co-Mn samples (Fig 2D and 2F), the flower-like structure overlaps because of the adjoining particles, as evidenced in the FESEM results. The overlapping of the flower-like structure can limit the cation intercalation in the electrode matrix and leads to low electrochemical performance. The presence of spherical particles can be attributed to the instantaneous nucleation at all available sites during the first electrodeposition of MnO_2_ step (Fig 1E). The development of various textures on the flower-like structure on top of MnO_2_ is influenced by the progressive nucleation from the second electrodeposition step. Previous studies [22–23] reported that progressive nucleation occurs on a larger number of active sites than instantaneous nucleation does, and it not only forms on the substrate surface but also on previously formed nuclei, resulting in the growth of compact grains. The growth of thicker, more compact grains in Ni-Mn and Ni-Co-Mn samples caused the overlapping of the particles.

**Fig 3:**
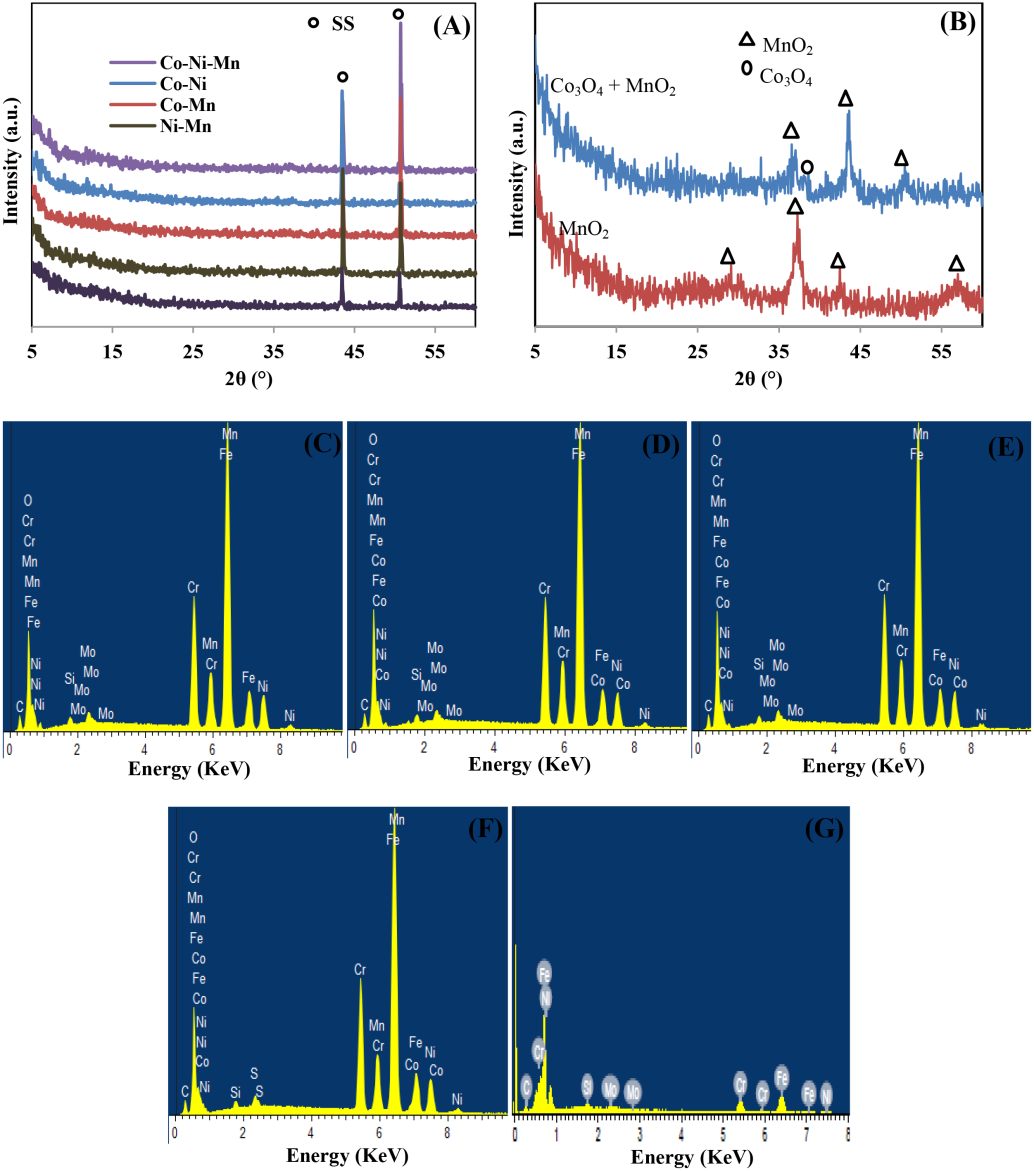
(A) XRD pattern of all deposited electrodes on top of SS, (B) XRD pattern of scraped off powder of MnO_2_ deposits (first electrodeposition) and scraped-off powder of Co-Mn deposits after the second electrodeposition step, EDX images of: (C) Co-Mn, (D) Ni-Mn, (E) Co-Ni-Mn, (F) Co-Ni, and (G) Empty SS.

The elemental identity of the deposited metal oxide on SS was confirmed by XRD. No significant peaks could be observed from the XRD patterns of all deposited electrodes on SS substrate (Fig 3A). This absence is attributed to the thin amorphous deposited sample [24]. Fig 3B shows the XRD pattern of scraped-off powder from Co-Mn deposits on SS, which was collected after the second electrodeposition step. The peaks at 2θ = 29.1°, 37.3°, 42.5° and 56.6° in the XRD pattern of deposited MnO_2_ from the first electrodeposition step confirm the formation of α-MnO_2_ with planes of (310), (211), (301), and (600), respectively (JCPDSNO.44-0141) [25]. A low intensity peak around 2θ = 38.5° corresponding to the (222) plane of Co_3_O_4_ (JCPDS No. 76–1802) [26] is detected at the point when the second layer composed of Co-Mn was deposited on the MnO_2_ particle layer, and other peaks at 2θ = 37.3°, 42.5°, and 50.6° belong to (211), (301), and (411) planes of α-MnO_2_ peaks [25]. The broad nature and weak relative intensity of the Co_3_O_4_ peak imply a small size of Co_3_O_4_ and the amorphous nature of deposits, which is feasible for a supercapacitor application [24, 27–28]. The elemental composition has been confirmed from EDX studies (Fig 3C to 3G). The presence of C, Cr, Fe, Si, Mo, and some element traces of Ni were detected from empty SS.

### Electrochemical studies

**Fig 4:**
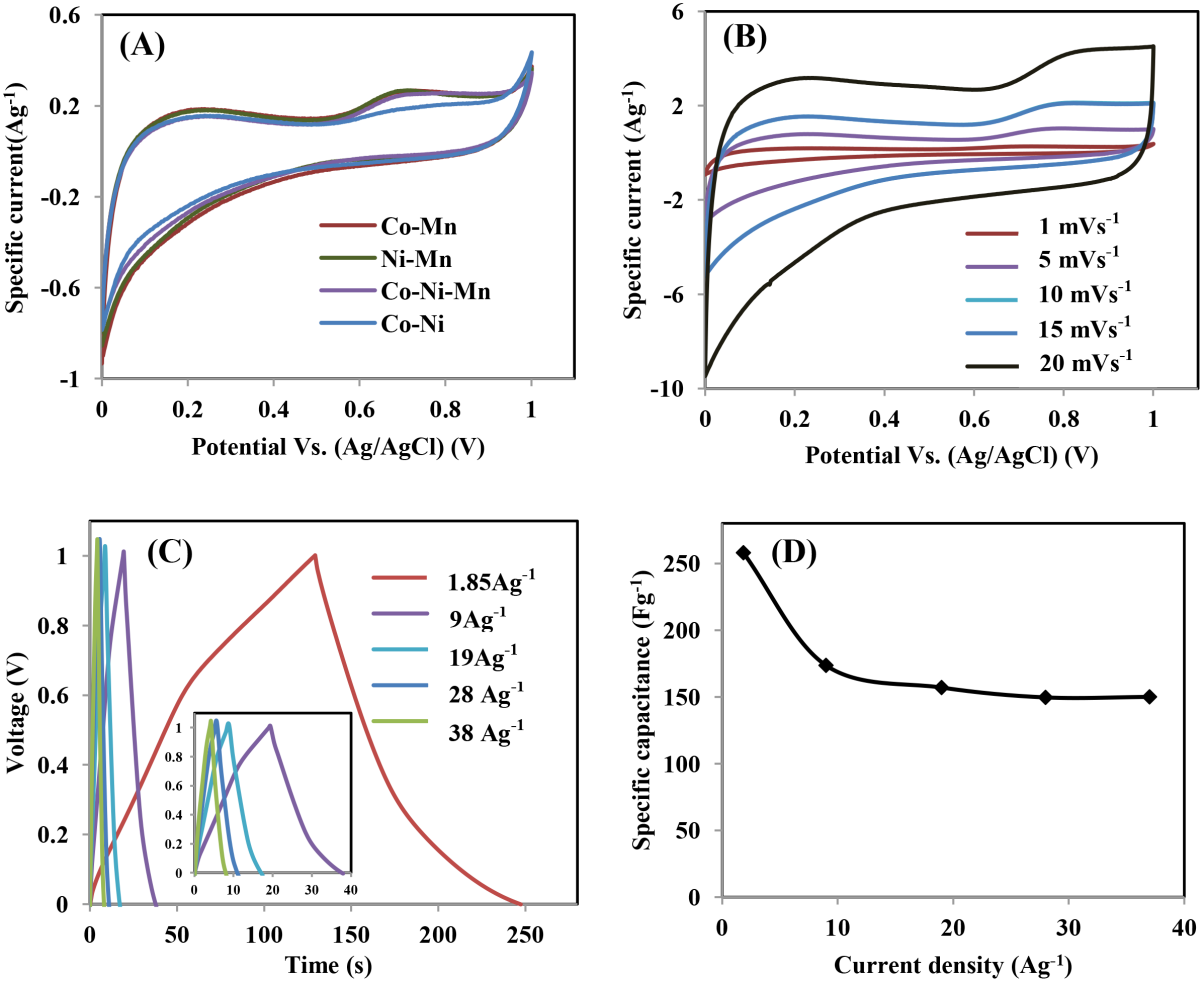
(A) CV curve of all deposited electrodes at 1 mVs^-1^, (B) CV curve of the best Co-Mn deposited electrode at different scan rates, (C) charge-discharge performance of Co-Mn electrode at different current densities in 0.5M Na_2_SO_4_ electrolyte, (D) the specific capacitance calculated at different current densities.

The electrochemical performance of the electrode materials can be influenced by the morphology of materials, so the prepared electrodes were subjected to cyclic voltammetry (CV), charge-discharge (CD), and impedance tests to investigate the effect of the second electrodeposited layer on the electrochemical performance of MnO_2_-based electrodes. The metal oxide electrode can store charges at the electrode/electrolyte interface, and a redox reaction takes place in the alkaline electrolyte. The cyclic voltammetry curves of all samples in a potential window of 0-1V using a 0.5 M Na_2_SO_4_ electrolyte at a scan rate of 1 mVs^-1^ are displayed in Fig 4A. CV curves show well-defined redox peaks indicating the significant contribution of faradic behaviour than the EDLC. The maximum area under the curve is obtained from the CV curves of the Co-Mn sample. The specific capacitance can be calculated from the CV curve with the following equation: Eq 1:

$$C=\;\frac{\int I\;dt}{\Delta E\;x\;m}$$ (1)

where *I* is the oxidation/reduction current, *dt* is time differential, *m* is the mass of active material and *ΔE* is the operating potential.

The calculated specific capacitance values for Co-Mn, Ni-Mn, Co-Ni-Mn, and Co-Ni samples are 186 Fg^-1^, 169 Fg^-1^, 156 Fg^-1^, and 153 Fg^-1^, respectively. Hence, the Co-Mn sample is identified as the best electrode in the present study. To further understand the capacity behaviour of this sample, the cyclic voltammetry curves were run at different scan rates, as illustrated in Fig 4B. As the scan rate increased, the specific capacitance reduced by 20% from 186 Fg^-1^ to 149 Fg^-1^ at a scan rate of 1 mVs^-1^ and 20 mVs^-1^, suggesting good electrode properties [29].

The charge-discharge measurement of the Co-Mn electrode was conducted from 0–1 V, using the same electrolyte (see Fig 4C). The specific capacitance from the discharging curve can be calculated using Eq 2:

$$C=\;\frac{I}{\frac{dE}{dt}\;x\;m}$$ (2)

where *I* is the discharge current, dE/dt is the change of discharge potential with the discharge time and *m* is mass of active materials.

The electrode can deliver a high specific capacitance of 258 Fg^-1^ at a current density of 1.85 Ag^-1^. Furthermore, a specific capacitance of 150 Fg^-1^ is still retained at a very high current density of 37 Ag^-1^, implying good specific capacitance retention behavior. (Fig 4D). The specific capacitance decreases as the increasing scan rate or current density reveals the minimum utilization of active materials. At higher scan rates or higher discharge constant currents, the Na^+^ ions reach only the outer surface of the electrode; the active material at the inner surface does not get fully involved in the electrochemical process [20, 28].

The best electrochemical performance is in deposited film with Co-Mn as a second layer and the success of this performance could be attributed to the following reasons: (1) the small particle size and no agglomeration between the particles increases the surface area of the electrode; (2) the thicker porous layer at the outer surface of the nanosphere facilitates fast electron transport paths for diffusion of the electrolyte into electroactive materials; (3) homogeneous distribution of the flower-like structure at the outer sphere surface might strongly improve the conductivity and contribute to high capacitance [28].

**Fig 5:**
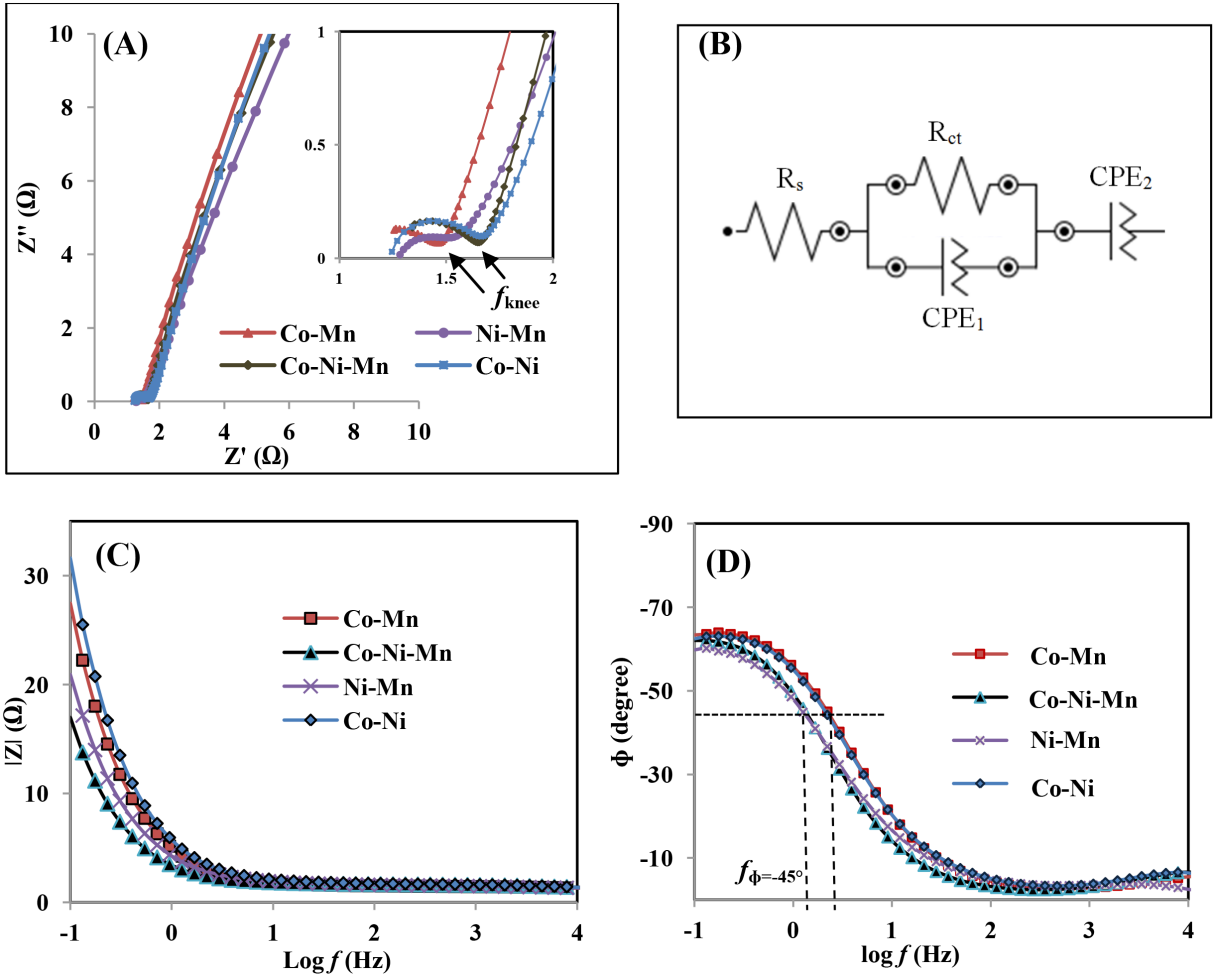
(A) Nyquist plots, (B) Simulation equivalent circuit, (C) frequency versus impedance magnitude, (D) frequency versus phase angle for all deposited samples.

Fig 5A shows an impedance plot of all deposits recorded in the 0.5M Na_2_SO_4_ electrolyte in a frequency range from 0.1 Hz to 100 kHz with a potential of 0V. The measured impedances were analyzed using a Nyquist plot, and the impedance data were employed to estimate quantities of the elements of the equivalent circuit by using Nova simulation software (Fig 5B). Generally, the impedance plot of supercapacitors can be divided into three regions according to processes, such as equivalent series resistance (ESR, R_s_), transfer resistance (R_ct_), and the Warburg diffusion region. The R_s_ value is obtained at the intercept of the high frequency of the impedance plots on the x-axis (Fig 5A) and represents the electrolyte resistance, intrinsic resistance of the electrode, and the contact resistance of metal oxide-current collector interfaces [30]. The R_s_ value of Co-Mn, Ni-Mn, Co-Ni-Mn and Co-Ni electrodes are 1.12, 1.27, 1.20, and 1.21Ω, respectively. The high-frequency arc corresponds to the charge transfer resistance (R_ct_), which is caused by a faradic reaction between electrode/electrolyte interface and can be determined by arc semicircle diameter [30–31]. It is was found that the sum of series resistance and transfer resistance (R_s_ + R_ct_) values of the Co-Mn electrode is lower than that of the Ni-Mn, Co-Ni-Mn, and Co-Ni electrodes, as shown in Table 2. Overall, the impedance of the Co-Mn electrode is smaller than others (evidenced by smaller R_s_ and R_ct_), which indicates that the cation insertion/extraction process into/from Co-Mn is more efficient than in the case of other electrodes, which might be due to the improved wettability properties. In the Warburg region, the smaller angle between the straight line portion at the low-frequency region and the real x-axis implies that there is a longer diffusion path length, which leads to a greater ion movement’s hindrance [32–33]. The biggest angle of Co-Mn indicates the faster ion diffusion of electrolytes than in Ni-Mn, Co-Ni-Mn, and Co-Ni electrodes, which implies a better electrochemical performance. The knee frequency (f_knee_) represents the maximum frequency that the stored energy can fully access. The determined f_knee_ value is as high as 356 Hz for Co-Mn and showed a good frequency response, indicating that this electrode has higher power capability and can be rapidly charged [34]. For further investigation, the electrode can be represented by an equivalent circuit, Fig 5B. In the circuit in this study, CPE_1_ and CPE_2_ were use to replace the double-layer capacity and Warburg diffusion impedance, respectively [35]. The simulation impedance values are close to the experimental values (see Table 2).

**Table 2:** Resistance and frequency values obtained from EIS for all deposited film.

Sample	R_s_ (Ω)	R_ct_ (Ω)	R_s_+ R_ct_ (Ω)	*f*_knee_ (Hz)	*f*_ɸ = -45°_ (Hz)	R_s_ (Ω) (simulation)	R_ct_ (Ω) (simulation)
Co-Mn	1.12	0.35	1.47	356	2.22	1.07	0.23
Ni-Mn	1.27	0.37	1.64	268	1.26	1.28	0.31
Co-Ni-Mn	1.20	0.46	1.67	202	1.27	1.19	0.47
Co-Ni	1.21	0.49	1.70	268	2.09	1.16	0.58

The bode plots shown in Fig 5C and 5D are the frequency dependence on impedance magnitude (|Z|) and phase angle (ɸ) of all deposited films. A typical capacitive characteristic of the electrode can be manifested from the analysis of these plots, which can be divided into three main segments [36–37]. In the first segment, at low frequency region (f<1Hz), all the samples exhibit a slope of ~ -1 in the plot log f versus (|Z|)) and the phase angle between -70° and -45° in the plot log f versus (ɸ), showing the capacitive characteristic. In the second segment, at intermediate frequency region, with a high frequency value at ɸ = -45°, represents a better capacitive response (Fig 5D). From this figure, it is seen that the deposited electrode of Co-Mn exhibits the highest frequency value of 2.22 Hz, indicating that it has a fast response time compared to other electrodes (see Table 2) [34] and results in a high specific capacitance of the Co-Mn electrode. This impedance study is in agreement with the CV and CDC results. In the third segment, at high frequency (f>10Hz), the phase angle starts to decrease from 20° to ~zero with increasing frequency.

### Electrolyte study

**Fig 6:**
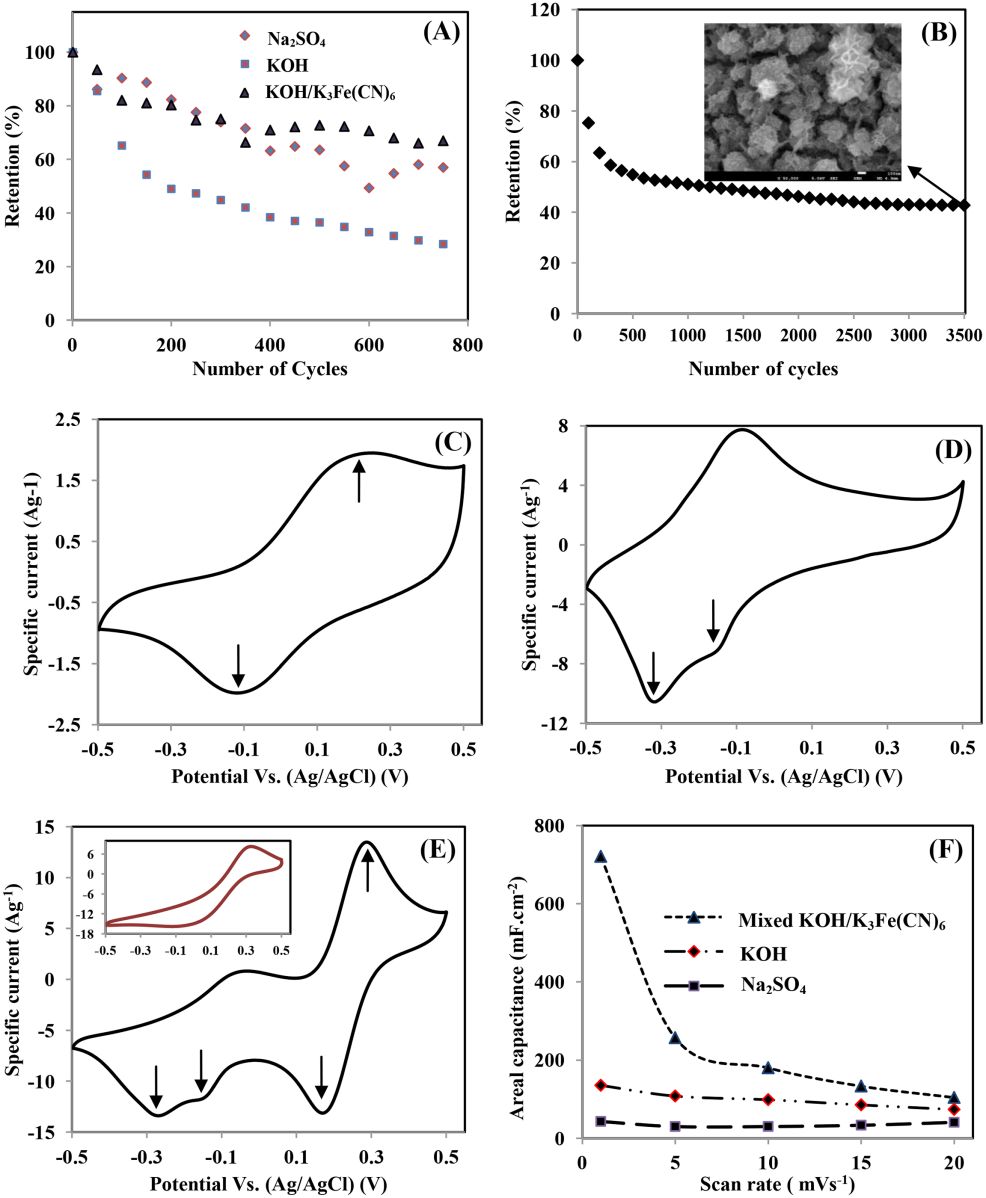
Electrochemical stability at voltage range of -0.5V 0 0.5V in: (A) three different electrolytes at a scan rate of 10 mVs^-1^ until 750 cycles, (B) mixed 0.5M KOH/0.04M K_3_Fe(CN)_6_ electrolyte at a scan rate of 100 mVs^-1^ until 3500 cycles and The FESEM images after 3500 cycles (insert), CV responses of Co-Mn in different electrolytes: (C) 0.5M Na_2_SO_4_, (D) 0.5M KOH, (E) mixed 0.5M KOH/0.04M K_3_Fe(CN)_6_ (inset: bare 0.04M K_3_Fe(CN)_6_ electrolyte) in voltage range of -0.5 V to 0.5 V at scan rate of 5 mVs^-1^, and (F) the specific capacitance per surface area of Co-Mn electrodes in three different electrolytes at varying scan rates.

To further investigate the performance of the Co-Mn electrode in different alkaline electrolytes, cyclic stability tests for 750 cycles at a scan rate of 10 mVs^-1^ were performed (Fig 6A). The specific capacitance retention of Co-Mn in Na_2_SO_4_, KOH and mixed KOH/K_3_Fe(CN)_6_ electrolytes after 750 cycles was 57%, 29%, and 67%, respectively. Low capacitance retention over 750 cycles could be attributed to high degradation of the electrode, resulting from the high current passed through during the cyclability test and volume loss of active materials [38]. In comparison with the Na_2_SO_4_ electrolyte, KOH is less stable for the Co-Mn electrode. However, the capacitance retention of the Co-Mn electrode in KOH is remarkably enhanced when 0.4M K_3_Fe(CN)_6_ is added to the KOH electrolyte. The retention test of the Co-Mn electrode was further studied at a higher scan rate of 100 mV s^-1^ in a mixed KOH/K_3_Fe(CN)_6_ electrolyte, as shown in Fig 6B. The specific capacitance retained 43% of initial capacitance value after 3500 cycles. However, the long term cycling test changed the morphology of the Co-Mn electrode (Fig 6B insert) and caused the loss of specific capacitance retention.

The cyclic voltammograms of the obtained Co-Mn electrode were recorded in 0.5M Na_2_SO_4_, 0.5 M KOH, 0.04M K_3_Fe(CN)_6_, and mixed 0.5M KOH/0.04M K_3_Fe(CN)_6_ electrolytes by sweeping the potential from -0.5V to 0.5V at a scan rate of 5 mVs^-1^, as shown in Fig 6C, 6D and 6E. In the case of the Na_2_SO_4_ electrolyte (Fig 6C), a CV curve with a well-defined pair of anodic peaks (A_0_) and a cathodic peak (C_0_) centered at +0.22 and -0.12 V (vs. Ag/AgCl) was observed. These peaks are corresponds to the redox reaction dominated by MnO_2_, according to Eqs 3–4 [4]:

$$MnO_{2}+\;H^{+}+\;e^{-}↔\;{\left(MnOOH\right)}_{surface}$$ (3)

or

$$MnO_{2}+\;K\;+\;e^{-}↔\;{\left(MnOOK\right)}_{surface}$$ (4)

(K: cations in electrolyte (i.e., Na^+^ or K^+^))

The KOH electrolyte is known to be a better electrolyte for Co_3_O_4_ materials than Na_2_SO_4_ aqueous solution, due to the higher OH^-^ concentration in the electrolyte solution. OH^-^ ions play an important role in Co_3_O_4_ reaction [19,39] during charging/discharging (Eq 5).

$$Co_{3}O_{4}+\;OH^{-}+\;H_{2}O↔\;3CoOOH\;+e^{-}$$ (5)

The combined contribution of MnO_2_-Co_3_O_4_ in the KOH electrolyte (Fig 6D), is supported by the appearance of C_0_ peaks at -0.14 and -0.31 V, according to reactions shown in Eqs 3–5. The increment of current response in the KOH electrolyte is also attributed to the K^+^ ion’s smaller cation radius (3.31Å) compared to Na^+^ ions (3.35 Å), as well as the higher conductivity of K^+^ ions (73 cm2/Ω mol) than Na^+^ ions (50 cm^2^/Ω mol). Easy passage of K^+^ ions into the electrode matrix during the charging process is achieved because it has a smaller radius and faster ion movements [40].

When KOH is replaced with mixed KOH/K_3_Fe(CN)_6_ electrolyte, as in Fig 6E, an additional pair of anodic peaks at +0.27V (A_02_) and a cathodic peak at +0.17V (C_02_) are detected, which can be attributed to the redox reaction of K_4_Fe(CN)_6_ to K_3_Fe(CN)_6_, and it is consistent with the CV plot of bare K_3_Fe(CN)_6_ electrolyte (insert picture) [39]. In this system, there are two types of charge storage reaction that could contribute to the capacitance. The first reaction is originated from the redox couple of [Fe(CN)_6_]^3-^ / [Fe(CN)_6_]^4-^ in the electrolyte (Eq 6). The second type of charge storage can be derived from the redox reaction in highly electroactive electrodes (Eq 7). The reaction can be written as follows [41]:

$$Redox\;electrolyte:\;{\left[Fe{\left(CN\right)}_{6}\right]}^{3-}+\;e^{-}↔\;{\left[Fe{\left(CN\right)}_{6}\right]}^{4-}$$ (6)

$$Redox\;electrode:\;M^{z+}↔\;M^{\left(z+n\right)+}+\;ne^{-}$$ (7)

Where M is the Co^2+^ or Mn^2+^ cations, and 1≤n≤z.

**Fig 7:**
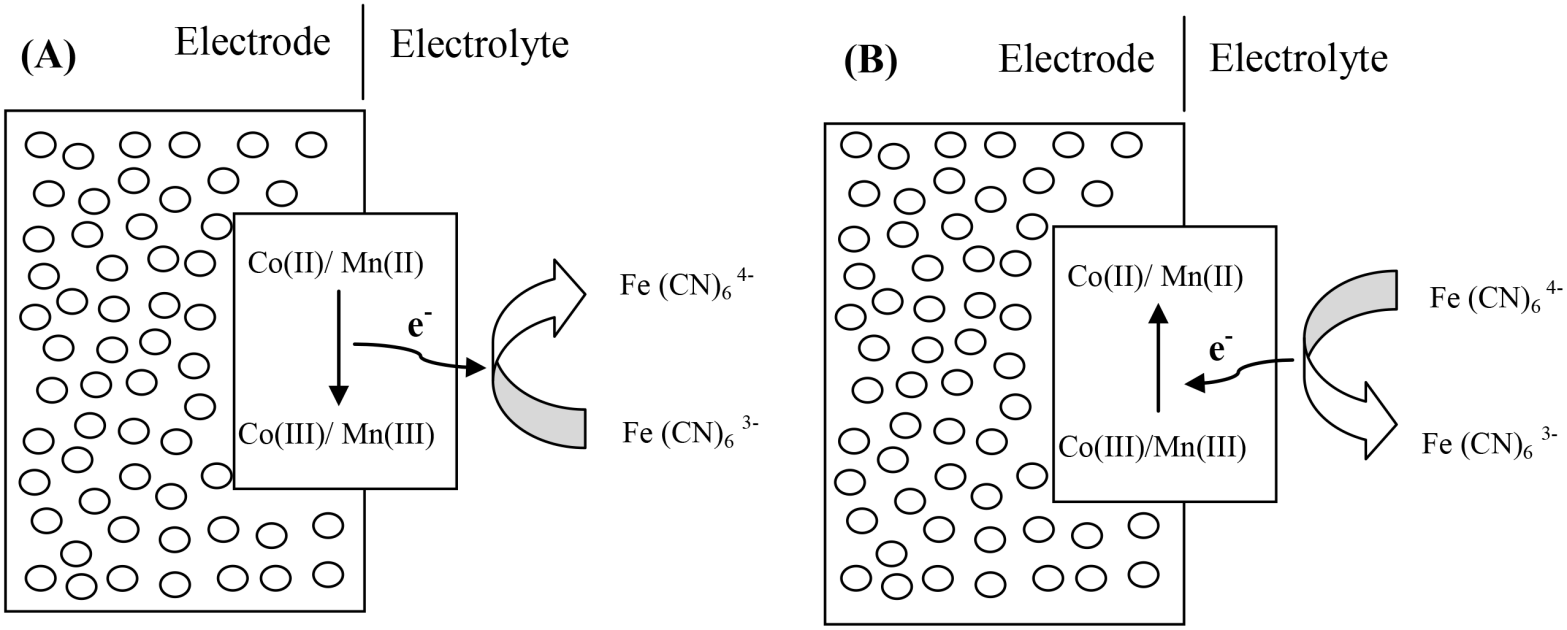
Schematic of the role of hexacyanoferrate (II) and (III) in the process of: (A) charge and (B) discharge of Co-Mn electrode.

Other than the electrode redox reaction of Co-Mn oxide in the KOH electrolyte, the hexacyanoferrate ions also play a role as “electron shuttles” in the charging/discharging process [42]. When the electrode is charged, [Fe(CN)_6_]^3-^ will accept the electron via the reduction of hexacyanoferrate (III) to (II), the hexacyanoferrate ions of which act as “electron carriers” (Fig 7A). When the reaction is reversible, the hexacyanoferrate ions act as “electron donors” and [Fe(CN)_6_]^4-^ returns to [Fe(CN)_6_]^3-^ will provide the electron for the transition process from Co(III) to Co (II) or Mn(III) to Mn(II) (Fig 7B). This performance helps the active material to lose and gain electrons smoothly and improves the capacitive performance [41].

Comparing all the curves, we see that the Co-Mn electrode in the mixed KOH/K_3_Fe(CN)_6_ electrolyte has a bigger area under the curve, implying high specific capacitance. The calculated specific capacitances from the CV at 5 mVs^-1^ are as follows: 210 Fg^-1^, 757 Fg^-1^, and 1658 Fg^-1^ for Na_2_SO_4_, KOH, and mixed KOH/K_3_Fe(CN)_6_ electrolytes, respectively. The areal capacitances of the Co-Mn samples in three electrolytes at different scan rates is shown in Fig 6F. The low scan rate results in a higher specific capacitance, due to the slow charging/discharging process in which the cation could access almost all available pores and materials was fully utilized [20]. The importance of K_3_Fe(CN)_6_ has been confirmed by the enhancement of specific capacitance and improvement of electrode stability, suggesting that the mixed electrolyte is the stable electrolyte for the Co-Mn electrode.

**Fig 8:**
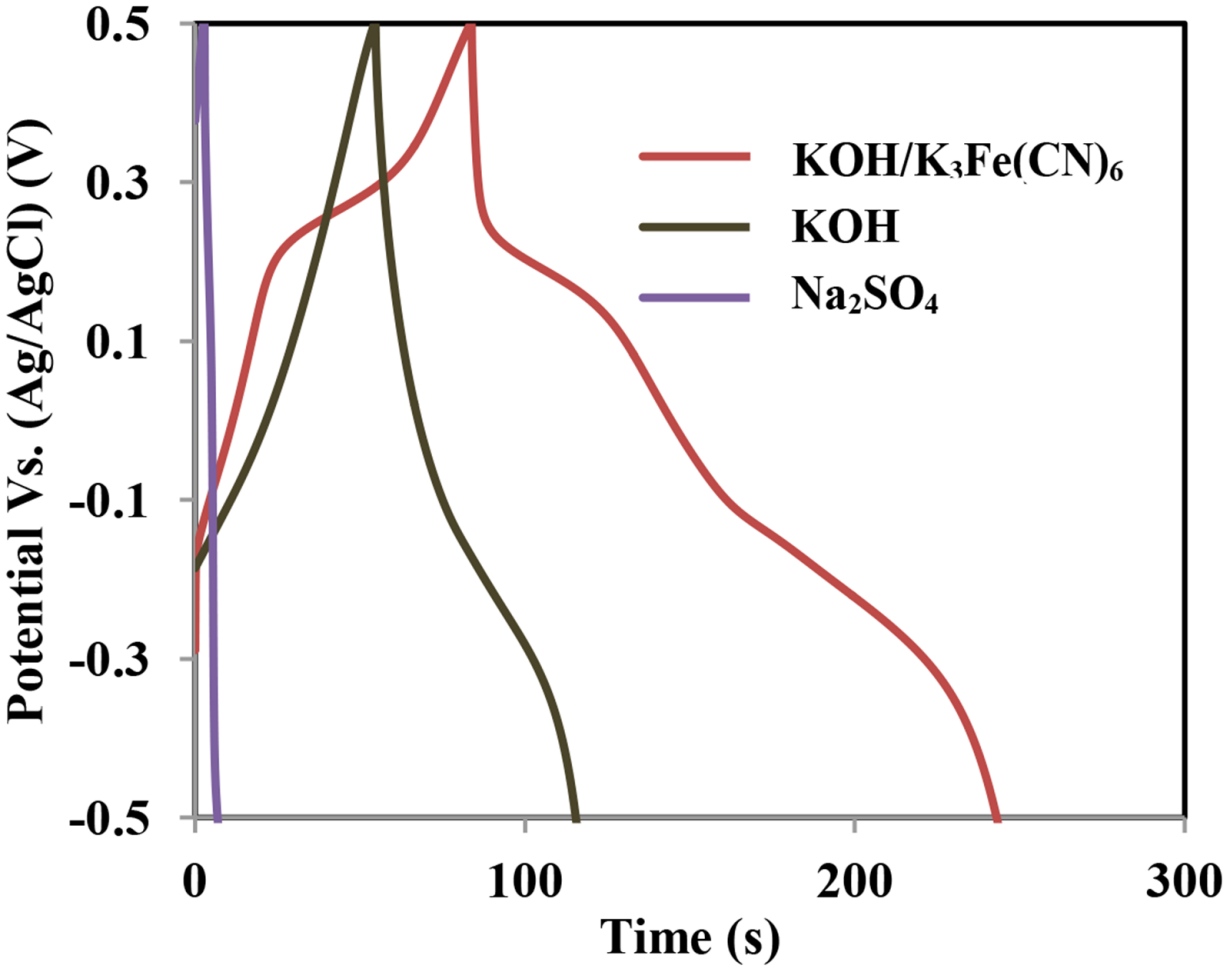
Charge-discharge profiles of Co-Mn electrode at current density of 10 Ag^-1^ in three different electrolytes.

Fig 8 illustrates the charge/discharge profiles of Co-Mn in Na_2_SO_4_, KOH, and mixed KOH/ K_3_Fe(CN)_6_ electrolytes at a current density of 10 Ag^-1^. The voltage range applied is from -0.5V to 0.5V. The charge/discharge profile displayed a slightly non-linear curve, which represented the pseudocapacitance characteristic resulting from the faradic reaction in the studied voltage range [43]. The specific capacitance, energy, and power densities calculated from the discharging curve for the mixed KOH/K_3_Fe(CN)_6_ electrolyte are 2222 Fg^-1^, 309 Whkg^-1^, and 73 kWkg^-1^ at a current density of 10 Ag^-1^. In the KOH and Na_2_SO_4_ electrolytes, the specific capacitances of 909 Fg^-1^ and 39 Fg^-1^ were obtained. The energy and power densities of Co-Mn electrodes in KOH and Na_2_SO_4_ electrolytes are 126 Whkg^-1^, 5 Whkg^-1^, 82 kWkg^-1^, and 24 kWkg^-1^, respectively.

### Conclusions

In summary, we have successfully fabricated a MnO_2_-based binder-free composite electrode material using the electrodeposition method. The composite electrode was obtained when a layer of secondary and ternary metal oxide containing Co, Mn, and Ni was ex situ deposited on a MnO_2_ particle layer by the same method. From the FESEM and TEM studies, we observed that the MnO_2_ particles were encapsulated by the second deposited metal oxide layer and had a strong influence on the electrochemical performance of the electrode. The capacitive performance of the Co-Mn electrode in the 0.5M Na_2_SO_4_ electrolyte exhibits the highest specific capacitance (285 Fg^-1^ with a current density of 1.85 Ag^-1^), the smallest R_s_ and R_ct_, and a high knee frequency of 356 Hz. The performance of the electrochemical capacitor in a three-electrode system employing the Co-Mn electrode in KOH and mixed KOH/K_3_Fe(CN)_6_ electrolytes confirms the importance of K_3_Fe(CN)_6_ in terms of specific capacitance enhancement and electrode stability. The calculated specific capacitance and energy density of the Co-Mn electrode in the mixed electrolyte was 2.2 x10^3^ Fg^-1^ and 309 Whkg^-1^ at a current density of 10 Ag^-1^. These statistics show Co-Mn electrode’s great potential for use as an electric energy storage device for hybrid vehicles.
